# Impact of donor-recipient ethnic matching on survival after lung transplantation in Italy

**DOI:** 10.1007/s13304-025-02371-2

**Published:** 2025-08-25

**Authors:** Alessandro Palleschi, Marco Schiavon, Paola Besani, Federico Rea, Daniele Diso, Federico Venuta, Chiara Catelli, Luca Luzzi, Matteo Marro, Massimo Boffini, Matteo Petroncini, Filippo Antonacci, Lorenzo Rosso, Mario Nosotti

**Affiliations:** 1 University of Milan, Milan, Italy; 2 University of Padua, Padua, Italy; 3 Sapienza University, Rome, Italy; 4 University of Siena, Siena, Italy; 5 Cardiac Surgery Division, Department of Surgical Sciences, City of Health and Science University Hospital, University of Turin, Turin, Italy; 6 IRCCSAzienda Ospedaliera Universitaria of Bologna, Bologna, Italy

**Keywords:** Lung transplantation, Ethnic mismatch, Donor-recipient matching, Health disparities

## Abstract

Disparities in access to and outcomes of lung transplantation have been extensively studied in North America, but little is known about these disparities in Europe. This study evaluates the impact of ethnicity on lung transplantation outcomes in Italy, including the role of ethnic mismatch between donor and recipient. We conducted a retrospective cohort study of patients undergoing lung transplantation between 2010 and 2020 in six Italian transplant centers. Demographic, clinical, and survival data were analyzed. The primary endpoint was survival following bilateral lung transplantation in Caucasian and non-Caucasian patients. Cox proportional hazards regression was used to identify factors associated with survival. Of the 959 patients studied, 93.4% were Caucasian and 6.6% non-Caucasian. Significant differences in access to transplantation were observed by ethnicity (*p* = 0.002). Non-Caucasian patients showed a trend toward lower unadjusted survival (*p* = 0.069), with significant differences linked to donor-recipient racial mismatch (*p* = 0.002). Cox regression identified recipient age, restrictive lung disease, education level, and donor-recipient mismatch as independent predictors of survival. Our study highlights disparities in lung transplantation outcomes linked to ethnicity and education level in Italy. Strategies to improve access and address donorrecipient mismatches could enhance equity in transplant care.

## Introduction

Lung transplantation is the standard of care for selected patients with end-stage lung disease. Since lung transplantation is a lifesaving procedure, the principles of utility, justice and respect for persons should be respected and guaranteed [1]. However, disparities in access and outcomes persist, influenced by socioeconomic status and ethnic background [2]. In particular, patients from ethnic minorities have historically experienced lower survival rates after organ transplantation in the United States [3]. Prior studies have demonstrated significant variation in lung transplant waitlist times and long-term outcomes based on a patient’s race [4–7]. Italy’s growing multicultural demographic includes significant increases in immigrant populations from Africa, Asia, and South America, which has implications for the availability and matching of organ donors and recipients of diverse ethnic backgrounds. Despite Italy’s universal healthcare system, ethnic, socioeconomic, and cultural disparities could pose barriers from referral to post-transplant outcomes, potentially affecting access to transplantation, survival rates, and long-term graft function [8]. One key factor that may influence these outcomes is donor–recipient ethnic mismatch, particularly due to differences in HLA allele frequencies among ethnic groups. Ethnic mismatch may exacerbate disparities by complicating optimal donor matching and increasing immunological risk. While the role of ethnic mismatch is well-documented in kidney and heart transplantation, [9–12] few studies have explored this factor in lung transplantation, especially in Europe. Most available data come from the United States, where disparities in healthcare access and transplant outcomes are more extensively studied [13, 14]. This study aims to address this gap by evaluating whether recipient ethnicity and donor–recipient ethnic mismatch affect survival after lung transplantation in Italy.

## Materials and methods

We conducted a retrospective cohort study of adult patients (≥ 18 years) with respiratory insufficiency who underwent lung transplantation between 2010 and 2020 in Italy. This timeframe was selected to ensure a sufficiently large cohort with adequate follow-up time. Demographic and clinical data were collected by clinical staff at six of the nine national lung transplant centers. Ethnicity designations were provided by each center and coded as Caucasian when appropriate, and as non-Caucasian for individuals from Asia, Africa, and South America. Educational level was categorized as'low'(primary and lower secondary school) and'high'(upper secondary school and university). Similarly, Lung Allocation Scores (LAS) were classified as'low'(≤ 45) and'high'(> 45). Continuous variables are expressed as mean and compared using *t*-tests. Categorical variables are presented as frequencies and compared using Chi-square tests. Time-to-event analysis was performed with Kaplan-Meier survival curves, and differences between groups were assessed using log-rank tests. Multivariate survival analysis was performed using Cox proportional hazards regression. Post-transplant survival analysis was performed using a causal inference approach based on inverse probability weighted regression adjustment (IPWRA). This method allows for a robust estimation of the average treatment effect (ATE) by accounting for potential imbalances between groups through inverse probability weighting and outcome regression. Two exposures of interest were analyzed: recipient ethnicity (Caucasian vs. non-Caucasian) and donor–recipient ethnic mismatch (matched vs. mismatched). Models were adjusted for clinically relevant confounders selected a priori. Results are presented as ATEs and potential outcome means (POmeans), each with 95% confidence intervals. Compared to traditional propensity score matching (PSM), the IPWRA method offers improved efficiency and consistency by combining treatment assignment and outcome modeling in a doubly robust framework, while preserving the full sample size [15]. HLA data were not available in our dataset, and ethnic mismatch refers strictly to donor-recipient ethnic differences.

All analyses were performed using STATA version 19 (StataCorp, College Station, TX, USA). The primary endpoint was survival following bilateral lung transplantation in Caucasian and non-Caucasian patients. Secondary analyses assessed whether ethnic mismatch independently affected outcomes. *p*-values <0.05 were considered statistically significant. The Milan Area B Ethical Committee approved the study (1680/2016).

## Results

Selecting 2015 as the representative year for the study period, the Italian population was 60,665,000, of which 54,865,000 were native and 5,800,000 (9.6%) belonged to other ethnicities. During the study period, 896 Caucasian patients (93.4%) and 63 non-Caucasian patients (6.6%) underwent bilateral lung transplantation at six Italian transplant centers. A Chi-square test indicated a significant association between ethnicity and access to lung transplantation (*X*^2^ = 9.924, *p* = 0.002). There were no differences in characteristics between the Caucasian and non-Caucasian cohort, except for the causes of lung disease requiring transplantation. The most notable difference was observed in cases of cystic fibrosis, which accounted for 34.8% of Caucasian patients compared to 15.9% of non-Caucasian patients. At the time of transplantation, there were no significant differences between the groups in the rates of ECMO support or invasive mechanical ventilation. Baseline donor characteristics were also well-distributed between the groups. The characteristics of patients in each ethnic group are presented in Table 1.

**Table 1:** Recipient and donor characteristics by ethnic group

	Total	Caucasians	Non-Caucasians	*p* value
Recipient number (%)	959	896 (93.4)	63 (6.6)	
*Recipient characteristics*				
Age, mean (SD)	44.6 (14.7)	44.6 (14.9)	43.9 (13.8)	0.695
Male, *n* (%)	553 (57.7)	523 (58.4)	30 (47.6)	0.097
High school, *n* (%)	500 (59.4)	473 (59.9)	27 (52.0)	0.307
LAS > 45, *n* (%)	171 (17.9)	160 (17.9)	11 (17.5)	0.936
*Diagnosis group, n (%)*				
Cystic fibrosis, *n *(%)	322 (33.6)	312 (34.8)	10 (15.9)	0.023*
Restrictive lung disease, *n* (%)	316 (32.9)	290 (32.4)	26 (41.3)	
Obstructive lung disease, *n* (%)	174 (18.1)	160 (17.9)	14 (22.2)	
Miscellanea, *n* (%)	104 (10.9)	96 (10.7)	8 (12.78)	
Pulmonary vascular disease, *n* (%)	43 (4.5)	38 (4.2)	5 (7.9)	
*Life support*				
No support, *n* (%)	859 (89.6)	805 (89.9)	54 (85.7)	0.612
Mechanical ventilation, *n* (%)	22 (2.3)	20 (2.3)	2 (3.2)	
ECMO, *n* (%)	78 (8.1)	71 (7.9)	7 (11.1)	
*Donor characteristics*				
DBD, *n* (%)	935 (97.5)	872 (97.3)	63 (100)	0.069
Age, mean (SD)	42.6 (15.1)	42.6 (15.1)	42.2 (15.7)	0.817
Male, *n* (%)	480 (50.0)	445 (49.7)	35 (55.6)	0.365
Smoke, *n* (%)	264 (27.6)	245 (27.4)	19 (30.2)	0.636
Diabetes, *n* (%)	25 (2.6)	25 (2.8)	0 (0)	0.416
Caucasians, *n* (%)	894 (93.2)	839 (93.6)	55 (87.3)	0.079

*DBD* donation after brain death; *DS* standard deviation; *ECMO* extra-corporeal membrane oxygenation; *LAS* lung allocation score; *n* number

*p < 0.05 indicates statistical significance

**Fig. 1:**
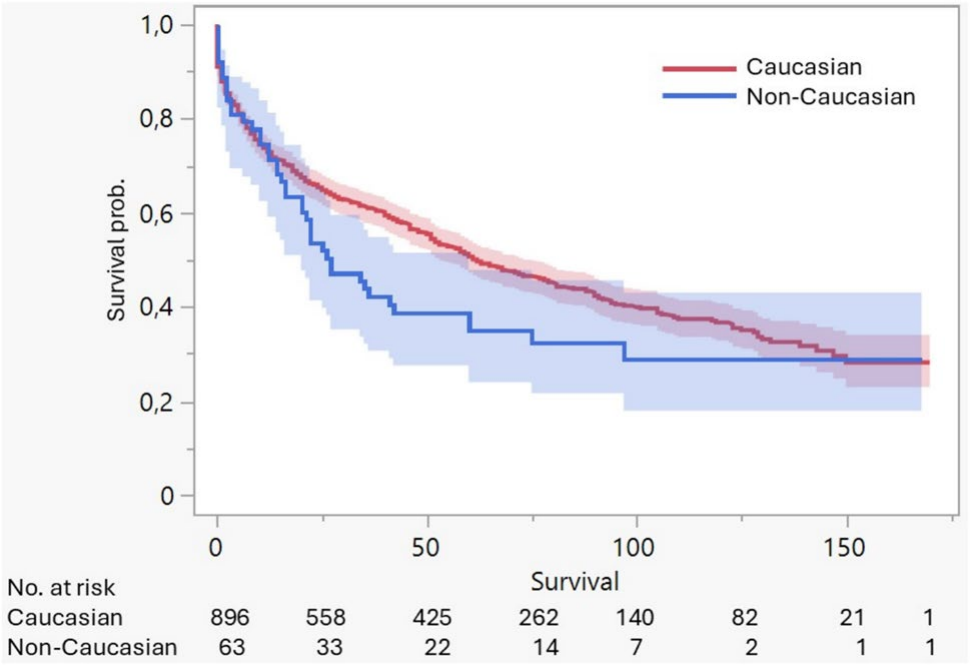
Kaplan–Meier survival curves comparing post-transplant survival between Caucasian and non-Caucasian recipients. Non-Caucasian recipients show a trend toward lower survival, but differences are not statistically significant (*p* = 0.069)

**Fig. 2:**
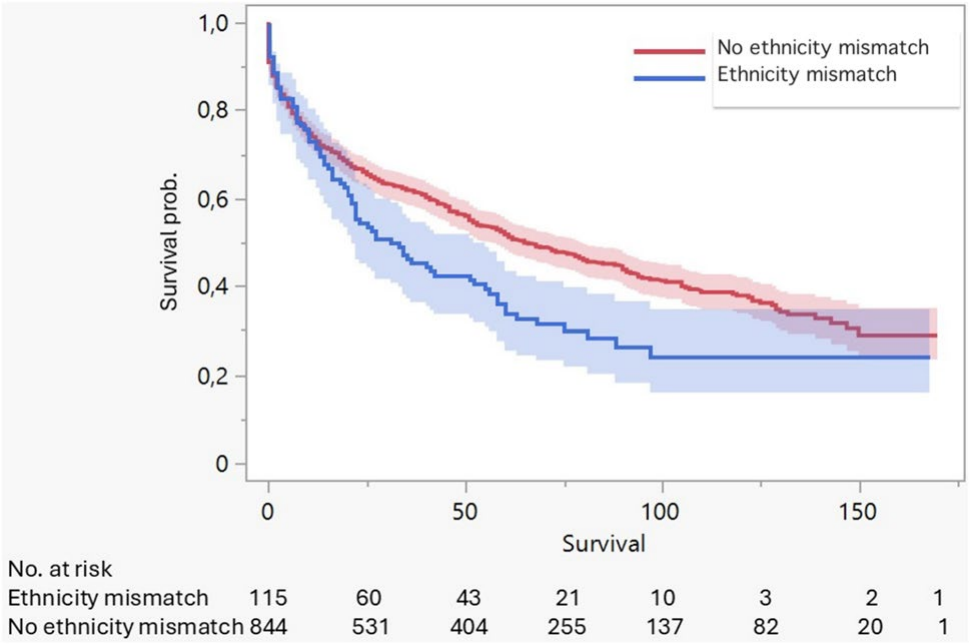
Kaplan-Meier survival curves for recipients with and without donor-recipient ethnic mismatch. Patients with ethnic mismatch exhibit significantly lower survival (*p* = 0.002)

During the study period, 545 post-transplant deaths were recorded. Non-Caucasian patients showed a tendency toward lower unadjusted survival compared with Caucasian patients, although the log-rank test did not reach statistical significance (*p* = 0.069) (Fig. 1). Overall, significant differences in unadjusted survival were observed by gender, education level, and diagnosis groups. Specifically, patients with higher educational levels had better survival than those with lower educational levels (mean: 86.8 versus 70.9 months). Finally, we recorded an ethnic mismatch between recipient and donor in 115 patients (12%) who underwent lung transplantation. Patients with an ethnic mismatch had significantly lower unadjusted survival compared to those without a mismatch (*p* = 0.002) (Fig. 2).

The Cox regression analysis did not indicate an increased mortality risk for non-Caucasian patients compared to Caucasian patients. Conversely, the following variables had a significant impact on mortality risk: recipient age (HR = 1.02), male gender of the recipient (HR = 1.24), recipient's restrictive pulmonary disease (HR = 2.18), recipient's higher education level (HR = 0.74), donor age (HR = 1.001), and donor smoking history (HR = 1.25). The univariable regression demonstrated an increased risk of death in patients with donor-recipient ethnic mismatch (HR = 1.44) (Table 2). By including the covariates significant in the univariable analysis in a multivariable regression model, the recipient's higher education level remained protective against mortality risk (HR= 0.80). Donor-recipient mismatch (HR = 1.36), recipient's restrictive pulmonary disease (HR = 1.37), and recipient age (HR = 1.01) also confirmed their negative impact on survival in the multivariable regression model (Table 3).

**Table 2:** Univariable logistic regression model for risk of death

Variable	HR (95% CI)	*p* value
*Recipient characteristics*		
Age	1.0208 (1.0145 to 1.0271)	< 0.0001*
Gender (male)	1.2477 (1.0495 to 1.4834)	0.0122*
Diagnosis group (ref. Cystic fibrosis)		
Miscellanea	1.8816 (1.3982 to 2.5321)	< 0.0001*
Restrictive lung disease	2.1784 (1.7567 to 2.7012)	
Pulmonary vascular disease	1.1736 (0.7342 to 1.8759)	
Obstructive lung disease	1.6791 (1.3047 to 2.1609)	
Education (high school)	0.7485 (0.6253 to 0.8959)	0.0016*
LAS (LAS > 45)	1.0693 (0.8592 to 1.3307)	n.s.
*Preoperative support (ref. none)*		
ECMO	0.8416 (0.6116 to 1.1580)	n.s.
Mechanical ventilation	0.9007 (0.4955 to 1.6371)	
Ethnicity (not Caucasian)	1.3320 (0.9720 to 1.8254)	n.s.
*Donor characteristics*		
Age	1.0077 (1.0020 to 1.0134)	0.0083*
Gender (male)	0.9683 (0.8185 to 1.1455)	n.s.
Smoke history (yes)	1.2505 (1.0427 to 1.4998)	0.0159*
Diabetes (yes)	0.7733 (0.4256 to 1.4048)	n.s.
Donor type (DCD)	0.7194 (0.3847 to 1.3453)	n.s.
Ethnicity (ref. not Caucasian)	1.3645 (0.9989 to 1.8641)	n.s.
*Donor-recipient mismatch*		
Height discrepancy	0.9995 (0.9918 to 1.0073)	n.s.
CMV mismatch (yes)	1.0233 (0.8024 to 1.3052)	n.s.
Ethnicity mismatch (yes)	1.4412 (1.1346 to 1.8307)	0.0028*

*CI* confidence interval; *CMV* cytomegalovirus; *DCD* donation after cardiocirculatory death; *ECMO* extra-corporeal membrane oxygenation; *HR* hazard ratio; *LAS* lung allocation score; *n.s* not significant; *ref.* reference

*p < 0.05 indicates statistical significance

**Table 3:** Multivariable logistic regression model for risk of death

Covariate	HR	95% CI	*p* value
Recipient age	1.0163	1.0092 to 1.0235	<0.0001*
High school (yes)	0.8034	0.6700 to 0.9635	0.0182*
Ethnicity mismatch (yes)	1.3633	1.0480 to 1.7734	0.0209*
Restrictive lung disease (yes)	1.3706	1.1215 to 1.6750	0.0021*

*CI* confidence interval; *HR* hazard risk

*p < 0.05 indicates statistical significance

In the IPWRA-adjusted survival model, the estimated average treatment effect (ATE) of Caucasian ethnicity on post-transplant survival was +69.3 days (95% CI – 34.0 to + 172.6; *p* = 0.189), indicating a trend toward longer survival among Caucasian patients. However, this difference did not reach statistical significance. The potential outcome mean (POmean) for non-Caucasian recipients was estimated at 119.8 days (95% CI 25.2 to 214.5), suggesting a wide variability in this subgroup. In the adjusted survival analysis using IPWRA, ethnic mismatch between donor and recipient was significantly associated with reduced survival. The estimated average treatment effect (ATE) for ethnic mismatch was –108.4 days (95% CI – 173.9 to –42.8; *p* = 0.001), indicating a substantial and statistically significant decrease in survival among patients experiencing donor–recipient ethnic mismatch. The potential outcome mean (POmean) for recipients without a mismatch was 202.6 days (95% CI 154.6 to 250.6), underscoring the clinical relevance of ethnic compatibility in lung transplantation outcomes.

## Discussion

Our analysis revealed a statistically significant underrepresentation of non-Caucasian patients among lung transplant recipients in Italy relative to their proportion in the general population. While this Chi-square test finding raises concerns about potential disparities in transplant access, the absence of epidemiological data on end-stage lung disease prevalence by ethnicity precludes definitive conclusions. Thus, this discrepancy should be viewed as a hypothesis-generating signal rather than conclusive evidence of inequity. Nevertheless, our findings align with previous Italian research indicating that immigrant and non-European populations may encounter structural and cultural barriers throughout the transplant pathway—including referral, evaluation, and post-operative care. Reported obstacles include language difficulties, challenges navigating the healthcare system, and problems establishing trust and continuity with providers [8]. Regarding post-transplant outcomes, we observed no statistically significant survival differences between Caucasian and non-Caucasian groups, although there was a trend toward poorer survival in the latter. Literature from other contexts indicates that both recipient and donor ethnicity can influence survival outcomes [16, 17]. Recent U.S. studies report significant disparities in 1- and 5-year mortality by ethnicity, also varying by gender and primary diagnosis [16].

A key novel finding in our study is the independent association between ethnic mismatch and increased mortality risk. Patients receiving lungs from donors of a different ethnicity had significantly worse survival in both Cox regression and IPWRA models. While European data on ethnic mismatch are scarce, our results are consistent with U.S.-based research showing that donor-recipient ethnic matching reduces mortality risk at multiple time points post-transplant [18, 19]. This supports growing evidence that ethnic mismatch is a clinically relevant factor warranting further investigation in Europe.

We also found that higher educational attainment positively associated with survival. Consistent with earlier Italian studies, migrants from non-EU countries generally have lower education levels and are less often referred for transplantation [8]. Education facilitates the ability to navigate the often complex and fragmented healthcare system, including scheduling appointments, accessing specialized services, understanding insurance or financial aspects, and advocating for one’s own care needs. This enhanced capacity can lead to earlier referrals, better pre-transplant preparation, and improved adherence to post-transplant care protocols, all of which contribute to better outcomes [20].

Our study has several limitations. Categorizing patients simply as Caucasian versus non-Caucasian limited ethnic granularity; small numbers within non-Caucasian subgroups precluded subgroup analysis. Moreover, the predominance of North American data in the literature complicates direct comparisons due to differing ethnic compositions. Although donor-recipient ethnicity mismatch was associated with lower survival, we lacked HLA mismatch data to assess whether this effect may reflect underlying immunological incompatibility. The relatively small sample of non-Caucasian patients underscores the need for larger multicenter European studies to deepen understanding. Finally, the retrospective multicenter design carries inherent biases related to inclusion criteria, variable treatment protocols, and missing data, limiting adjustment for confounders. Nonetheless, such studies remain valuable for hypothesis generation.

Reducing disparities in lung transplantation access and outcomes requires a multifaceted approach. Implementing culturally sensitive educational initiatives tailored to minority populations can help address the unique barriers they might face, such as language differences, and thereby improve access to transplant programs [21]. Additionally, raising awareness among healthcare providers about the importance of ethnic matching is critical. Recently, Cantu et al. proposed incorporating recipient race into allocation algorithms, exemplified by the Lung Donor Risk Index, which considers race alongside A and DR allele mismatches [22].

## Conclusions

In conclusion, our study is the first in a European country to evaluate the effect of ethnic mismatch on lung transplant survival, confirming its significant association with increased mortality and negative impact on survival. Given the differences in ethnic composition between North America and Europe, further studies are needed to identify the socio-economic and biological factors specific to lung transplantation in European.

## Data Availability

The datasets generated during the current study are not publicly available due to patient privacy and ethical restrictions, but are available from the corresponding author on reasonable request and with permission of the participating transplant centres.
